# The Therapeutic Potential of Medicinal Foods

**DOI:** 10.1155/2014/354264

**Published:** 2014-04-17

**Authors:** Nelvana Ramalingum, M. Fawzi Mahomoodally

**Affiliations:** Department of Health Sciences, Faculty of Science, University of Mauritius, 230 Réduit, Mauritius

## Abstract

Pharmaceutical and nutritional sciences have recently witnessed a bloom in the scientific literature geared towards the use of food plants for their diversified health benefits and potential clinical applications. Health professionals now recognize that a synergism of drug therapy and nutrition might confer optimum outcomes in the fight against diseases. The prophylactic benefits of food plants are being investigated for potential use as novel medicinal remedies due to the presence of pharmacologically active compounds. Although the availability of scientific data is rapidly growing, there is still a paucity of updated compilation of data and concerns about the rationale of these health-foods still persist in the literature. This paper attempts to congregate the nutritional value, phytochemical composition, traditional uses, *in vitro* and *in vivo* studies of 10 common medicinal food plants used against chronic noncommunicable and infectious diseases. Food plants included were based on the criteria that they are consumed as a common food in a typical diet as either fruit or vegetable for their nutritive value but have also other parts which are in common use in folk medicine. The potential challenges of incorporating these medicinal foods in the diet which offers prospective opportunities for future drug development are also discussed.

## 1. Introduction

With the epidemic of chronic diseases and associated pathological complications, health has become the forefront of scientific research for finding novel foods and strategies to tackle such public health burden. The past years have witnessed significant challenges in the traditional concepts of nutrition and pharmaceuticals. Indeed, the classical notion of “adequate nutrition,” that is, a diet that provides nutrients in sufficient quantities to satisfy particular organic needs, is being gradually replaced by the concept of “optimal nutrition.” This concept portrays food components as having the potential to promote health, improve general well-being, and reduce the risk of developing certain illnesses [1]. Hence, while attempting to unravel the various mechanisms by which food provide protection to the body, health experts have identified the presence of a plethora of bioactive compounds which are referred to as phytochemicals. This was the thrust that drove the conception of the term “functional food,” also expressed in a variety of terms like “pharmafoods,” “medifoods,” “vitafoods,” or “medicinal foods” [2]. Medicinal food plants may be defined as those food plants whose consumed parts receive recognition as therapeutic either in traditional medicine, ethnomedicine, or biomedicine [3].

**Figure 1:**
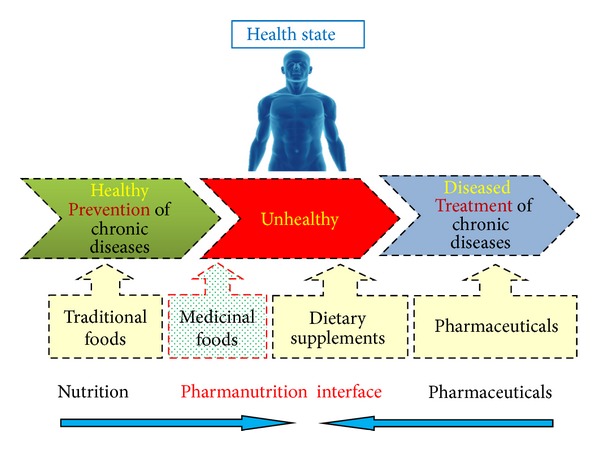
Pharmanutrition interface.

A holistic approach of the concept of medicinal foods was drawn from the study that foods are not intended to satisfy hunger and only provide essential macro- and micronutrients to the body but also to supply it with bioactive ingredients that aid to decrease nutrition-related diseases and ensure physical and mental well-being [4]. In contrast, nutraceutical has been defined as “food or part of food that provide medical or health benefits, including the prevention and treatment of disease” [5]. The main difference is that nutraceuticals can be consumed in a nonfood matrix form as pills, capsules, or tablets, whereas functional or medicinal foods are taken as part of a normal food pattern [6]. The amount of intake and form of the medicinal food should be as it is normally expected for dietary purposes. Functional foods were first launched in Japan in the early 1980s as a food category called Foods for Specific Health Use (FOSHU) [4]. To date, there is no real unanimous definition for functional food. However, the EU project, Functional Food Science in Europe (FUFOSE), has come forward with the following statement: “A food can be regarded as functional if it is satisfactorily demonstrated to affect beneficially one or more target functions in the body, beyond adequate nutritional effects, thus either improving the general physical conditions or/and decreasing the risk of the generation of diseases” [4]. In contrast, pharmaceuticals or drugs are especially formulated and developed to treat, cure, or prevent disease, which under normal conditions do not form part of our physiology [7]. Also, the pharmaceuticals have higher potency or biological activity compared to the phytochemicals which are normally ingested in insignificant amounts in diet and exert physiological effects only after long term use [6]. Therefore, a medicinal food can be considered to position itself between traditional foods and pharmaceuticals, termed as the “Pharmanutrition interface” (Figure 1) [8].

## 2. The Renaissance of Food Plants as Therapeutic Agents

Records of the use of plants for their therapeutic activities existed as early as from the Middle Paleolithic age [9]. From that point onwards, the value of this therapeutic approach has long been supported by traditional medicinal systems like Ayurveda, Unani, and Traditional Chinese Medicine. As defined by the World Health Organization (WHO), “traditional medicine is the sum total of knowledge, skills and practices based on the theories, beliefs and experiences indigenous to different cultures that are used to maintain health, as well as to prevent, diagnose, improve or treat physical and mental illnesses” [10]. Through generations, knowledge about botanicals and the savoir faire of preparing remedies have allowed humans to recognise the prophylactic benefits of certain plants and rely on their traditional* materia medica *for their healthcare needs [11]. Plants were administered mostly in their crude forms as infusions (herbal teas), tinctures (alcoholic extracts), decoctions (boiled extracts), and syrups (extracts of herbs made with syrup or honey) or applied externally as poultices, balms, and essential oils [11].

Among the earliest complete records which highlighted the use of over seven hundred herbs or plants as supplements were the Egyptian hieroglyphics. The Papyrus Ebers noted some of the herbs which are still in use today among which is* Aloe vera *L. [12]. A strong connection existed between food and pharmacology for maintaining health and treating various ailments. Indeed, Hippocrates, 500 BC stated the following: “Let food be your medicine and medicine be your food.” For example, spices which normally are used as flavour or taste enhancers in food were described as “influencers of body metabolism” [13]. Traditionally used in Indian cooking, turmeric (*Curcuma longa*) contains the active ingredient curcumin which is considered to have antioxidant, anti-inflammatory, and anticarcinogenic properties [14]. This is thought to be mediated through inhibition of several cell-signalling pathways, inhibition of enzymes such as cyclooxygenases and glutathione S-transferases, immunomodulation, and effects on angiogenesis and cell-cell adhesion [15]. Garlic (*Allium sativum*), which can be eaten raw or cooked, contains allicin. It has been documented to have LDL cholesterol lowering effects while increasing HDL levels, antihypertensive effects, and caused improved circulation [16]. Peppermint (*Mentha piperita*) has long been used to treat digestive problems like bloating, abdominal distension, and difficulty in evacuation due to its smooth muscle relaxant effect [17], while thyme (*Thymus vulgaris*) and sage (*Salvia officinalis*) of the Lamiaceae family were used to cure spasmodic gastric-intestinal complaints, cough, bronchitis, laryngitis, and tonsillitis and used as a vermifuge in ancient Egypt [18].

With rapid advances in pharmacological research, active ingredients from plants served as prototype molecules for possible development of novel drugs, with aspirin being first produced in 1897, derived from salicylic acid [19]. Consequently, this discovery ushered in an epoch of pharmaceutical development, where most ailments were treated with synthetic drugs. Until recently, the pharmaceutical industry has been faced with a “research drought” [20]. The unprecedented challenges are due to factors like the rising costs of research and development (R&D) in drug discovery and development, concerns about the integrity and transparency of the industry, and the strict regulations exerted by the Food and Drug Administration (FDA) in drug approval [21]. Coupled with this, there has been the swelling of the devastating scourge of chronic diseases worldwide, accounting for 80% of deaths among low and middle income countries [22] as well as a rampant spread of drug resistant pathogens causing infectious diseases. For example, diabetes mellitus and its pathological complications are costly to manage both for affected individuals and healthcare systems around the world. Much resource has been invested in the screening of antidiabetic agents in the past decades. As the knowledge of heterogeneity of these diseases increases, many people are shifting back to natural products. Natural products have served as a major source of drugs such that they contribute to about half of the armamentarium of pharmaceuticals in use today. On the other hand, more people are aiming towards the concept of self-care and believe that natural foods are associated with fewer side effects and consequently safer for use [23]. Their uses for health management has stood the test of time and are often considered relatively cheaper compared to synthetic drugs [24].

The past decade has witnessed an explosion of clinical research to show specifically what health benefits individual foods can offer, identifying the various nutrients and phytochemicals associated with these benefits and how they can be incorporated in the diet. One of the major issues of the WHO was to deepen investigation of plants as a promising source of therapies for human disease management [25]. Rationally designed polyherbal preparations are progressively being developed as alternative for multitarget therapeutic and prophylactic usage. This has resulted in growing lines of evidence to show that old molecules are finding new applications through a better understanding of traditional knowledge and clinical observations [26]. Till date, a miscellany of phytochemicals has been identified in medicinal plants to have versatile profile of effectiveness [24]. One sole plant may, for example, contain bitter substances that stimulate digestion, anti-inflammatory compounds, polyphenols that can act as an antioxidant, and venotonics, antibacterial, and antifungal tannins that perform as natural antibiotics [26]. In certain cases, when a combination of medicinal foods or extracts is consumed at the same time or mixed in appropriate formulation, the therapeutic effects could be a result of total sum of different classes of compounds present within the foods [27]. Indeed, there have been reports highlighting that intake of whole medicinal food which have resulted in significantly better outcomes compared when an equivalent dose of single isolated active ingredient was given. Thus, it can be argued that synergism can occur when two or more compounds interact in ways that mutually enhance, amplify, or potentiate each other's effect [28].

The present paper reviews common medicinal food plants which appeared in the literature with potential for the management of chronic diseases like diabetes mellitus, hyperlipidemia, or cancer and infectious illnesses. An attempt has also been made to congregate the nutritional value, phytochemical composition, traditional uses, and* in vitro* and* in vivo* studies from various common medicinal food plants (edible and nonedible parts) used among various populations worldwide for the management of both chronic noncommunicable and infectious diseases. The different plants included were based on the criteria that they are consumed as a common food in a typical diet as either fruit or vegetable for their nutritive value but have also other parts which are in common use in folk medicine. Another inclusion criterion was those food plants that came from different families which have previously gained scientific momentum and demonstrated to have medicinal virtues against panoply of ailments. Thus, focus was laid on Annonaceae, Moraceae, Myrtaceae, Cucurbitaceae, Moringaceae, Solanaceae, and Punicaceae families from which important phytochemicals have been isolated and offer prospective opportunities for future drug development.

### 2.1. *Annona squamosa *Linn. (Annonaceae): Sugar Apple or Custard Apple

*Annona squamosa*, also known as sugar apple or custard apple, is a small tropical tree which is native to South America and distributed throughout India and other tropical countries. The ripe fruit pulp contains around 88.9–95.7 g calories where the sugar content is 14.58%, amino acid lysine (54–69 mg), carotene (5–7 IU), and ascorbic acid (34.7–42.2 mg) [29]. The various chemical constituents isolated from leaves, stems, and roots of the plant include anonaine, aporphine, coryeline, isocorydine, norcorydine, and glaucine [30]. Folkloric record reports its use as an insecticidal and antitumor agent [31], antidiabetic [32], antioxidant, antilipidemic [33], and anti-inflammatory agent [34] which may be characterized due to the presence of the cyclic peptides [35]. An infusion with 2 handfuls of fresh leaves in 1 L of water is prepared to fight against heart failure and palpitations (1 cup after meal). This infusion is also effective for proper digestion and has antispasmodic activities [36]. The seeds are reported to have antiparasitic activities (against lice). A cream consisting of 3 cl bee wax, 12 cl almond oil, 3 cl coconut oil, 6 cl of water, 6 cl glycerin, and 1 handful of crushed* A. squamosa *seeds is prepared and heated over a water bath for 3 hours before applying to the hair [36]. In India the crushed leaves are applied on ulcers and wounds and a leaf decoction is taken in cases of dysentery [35]. In Aligarh district of Northern India, villagers used to consume a mixture of 4-5 newly grown young leaves of* A. squamosa *along with black pepper (*Piper nigrum*) for management of diabetes. It is documented that this may ensure up to 80% of the positive results with continued therapy [35]. The bark decoction is given as a tonic and to halt diarrhea. Throughout tropical America, a decoction of the leaves is imbibed either as an emmenagogue, febrifuge, tonic, cold remedy, digestive, or to clarify urine. The leaf decoction is also employed in baths to alleviate rheumatic pain [29].

The effect of aqueous and organic extracts from defatted seeds of* A. squamosa *was studied on a rat histolytic tumour cell line, AK-5. Both organic and aqueous extracts caused significant apoptotic tumour cell death with enhanced caspase-3 activity and downregulation of antiapoptotic genes Bcl-2 and Bcl-xl [37].

Chen et al. [38] have recently identified and quantified two main compounds, namely, annonaceous acetogenins from the ethanol extract of* A. squamosa* seeds. The extract was reported to exhibit an antitumor effect against H_22_ tumor cells line. Bullatacin, a bistetrahydrofuran annonaceous acetogenin is recognized as the most potent inhibitor of the mitochondrial respiratory chain complex I and was observed to be 300 times more active than taxol* in vivo* [39]. Water extracts of* A. squamosa *leaves also possess antioxidant activity as shown by increased activities of scavenging enzymes such as catalase, superoxide dismutase, reduced glutathione, and malondialdehyde levels present in various tissues [37]. Administration of the hot-water extract of leaves of* A. squamosa* at a dose 300 mg/kg body weight for 12 weeks to nephrectomized rats resulted in a significant decrease in the plasma urea and creatinine values with even partial restoration to normal values along with a significant rise in the activity of superoxide dismutase. Thus, custard apple shows potential for amelioration of renal failure [40]. Administration of the aqueous extract of the leaves also improved the activities of plasma insulin and lipid profile and reduced the levels of blood glucose and lipid peroxidation, indicating that the high levels of triglyceride and total cholesterol associated with diabetes can also be significantly managed with the extract [41, 42]. When petroleum ether, ethyl acetate and alcoholic extracts of* A. squamosa *fruit peel were administered orally (250 mg/kg body weight) for 21 days; the extracts showed a significant decrease of blood glucose level and lipid profile on streptozotocin (STZ) induced diabetic rats when compared to untreated diabetic control group [43].* A. squamosa* were also found to promote increased enzymatic (catalase, superoxide dismutase, glutathione peroxidase, and glutathione S-transferase) and nonenzymatic (vitamin E and ascorbic acid) antioxidants levels and nitric oxide levels in wound tissues for better wound repair mechanism in normal and diabetic rats [44].

The chloroform, petroleum ether, and ethanol extracts of custard apple also demonstrated important antimicrobial properties against the gram positive microorganisms such as* Bacillus subtilis, Bacillus cereus*,* Bacillus megaterium*,* Staphylococcus aureus*, and* Sarcina lutea* and the gram negative bacteria such as* Escherichia coli*,* Shigella dysenteriae*,* Shigella shiga*,* Shigella flexneri*,* Shigella sonnei*,* Salmonella typhi*,* Pseudomonas aeruginosa*, and* Klebsiella* spp. [35]. Similar results were also reported from the methanolic extracts of custard apple [45].

### 2.2. *Artocarpus altilis *Parkinson Fosberg (Moraceae): Breadfruit

*Artocarpus altilis*, also known as breadfruit is a pantropical tree widely distributed throughout Southeast Asia and most Pacific Ocean islands. It is a staple crop and a primary component of traditional agroforestry systems in Oceania [46]. The nutritional composition of breadfruit varies among cultivars. The fresh fruit contains around 22.8–77.3 g of carbohydrates (among which 68% is starch) calcium (15.2–31.1 mg), potassium (352 mg), phosphorus (34.4–79 mg), and niacin (0.81–1.96 mg). It is estimated that 2 cups (500 g) of boiled breadfruit at lunch and dinner provide around 25 g of fibre, but a similar serving of white rice provides only 6.8 g. A wide range of provitamin A carotenoid levels were found in breadfruit cultivars; some containing very high levels of 295–868 mg/100 g in breadfruit edible portion [47]. The essential amino acids that were found in the greatest amounts were leucine (605 mg) and lysine (799 mg) during the ripe developmental stage. The essential fatty acids detected at the highest concentration in ripe fruits were linoleic acid (0.15 mg) and linolenic acid (2.13 mg) [48]. Breadfruit can be consumed ripe, boiled fried, or grilled and is now commonly used to replace starchy vegetables, pasta, or rice. In the Indian culture, it is usually cooked with a blend of spices [47].

Breadfruit is a rich source of prenylated phenolic compounds such as geranylated flavones. For instance, artocarpin, isolated from an extract of heartwood of* A. altilis*, possesses potent *α*-reductase inhibitory effect and superoxide anion-scavenging activity [49]. Cataplasm of the fruits is effective against rheumatic and muscular pain, while decoction of the leaves has been documented to be a good antidote against ingestion of toxic fish [36]. The native people of Indonesia and Pacific islands use the fruit pulp as liver tonic, against liver cirrhosis and hypertension [50]. Most chitin-binding lectins have shown antifungal activity against phytopathogenic species, since chitin is the key component of the cell wall of these microorganisms. They have shown to affect fungal growth and development, disturbing the synthesis and/or deposition of chitin in the cell wall [51, 52]. The isolated chitin-binding lectins from the seeds of* A. altilis*, frutackin, promoted hemagglutination and growth inhibition against fungi* Fusarium moniliforme *and* Saccharomyces cerevisiae in vitro *[53].

Ethyl acetate and methanolic extracts of* A. altilis* have also demonstrated potential for significant antibacterial activities against various pathogenic organisms like* Staphylococcus aureus*,* Pseudomonas aeruginosa*,* Streptococcus mutans*, and* Enterococcus faecalis* [54]. Flavonoids present, inhibited 5-lipoxygenase of cultured mastocytoma cells* in vitro *[47] and prenylflavonoids showed antinephritis activity* in vivo *[55]. Aqueous extract of the leaves of* A. altilis* was found to exhibit negative chronotropic and hypotensive effects through *α*-adrenoceptor and Ca^2+^ channel antagonism and cause moderate inhibition of cytochrome P450s (CYP3A4 and CYP 2D6) [56]. A body of evidence suggests that advanced glycation endproducts (AGEs) play critical roles as pathogenic mediators of almost all diabetes complications, typically clustered as micro- or macrovascular lesions [57]. Diabetic inflammation is triggered by the engagement of the receptor for advanced glycation end products (RAGE) by AGEs on the surface of monocytes which subsequently induce the production of cytosolic reactive oxygen species and NF-*κ*B activation to promote the release of proinflammatory cytokines and facilitates the transformation of monocytes into macrophage. Recently, Lin et al. [58] have evaluated the effects of the geranyl flavonoid derivatives isolated from the fruit on the human THP-1 monocyte (THP-1) activation stimulated by S100B, a ligand of RAGE. It was found that addition of the geranyl flavonoid derivatives of breadfruit inhibited the S100B-induced THP-1 morphological characteristics of inflammation. The geranyl flavonoid derivatives inhibited S100B-induced reactive oxygen species generation, mRNA expression of inflammatory mediators (COX-2, TNF-*α*, and IL-6) [58].

### 2.3. *Artocarpus heterophyllus* Lam. (Moraceae): Jackfruit

*Artocarpus heterophyllus *is a tropical tree native to Western Ghats of India, Malaysia, and also found in Central and Eastern Africa, South-eastern Asia, the Caribbean, and many Pacific Islands [59]. Depending on the variety of the jackfruit, the proximate nutritional composition per 100 g of ripe fruit has been shown to vary. The ripe fruit has a proximate energy value of 88–410 KJ and principally contains 1.2–1.9 g of protein, 0.1–0.4 g of fat, 16–25.4 g of carbohydrates, 175–540 IU of vitamin A, 7–10 mg of vitamin C, 20–37 mg of calcium, 38–41 mg of phosphorus, and 191–407 mg of potassium [60]. Reports suggest that almost all parts of the jackfruit tree are of use in the preparations of various Ayurvedic and Unani medicines [61, 62] and ripe fruits are consumed to prevent excessive formation of bile, to develop flesh, phlegm, to strength the body, and increases virility [60]. Aqueous extracts of mature leaves of* A. heterophyllus* are used by traditional medical practitioners in Sri Lanka and India for the treatment of diabetes [63]. Roots useful in treating various skin diseases, asthma, and diarrhea. An ash produced by burning bark is supposed to heal abscesses and ear problems [61]. The infusion of mature leaves and bark is supposed to be effective in the treatment of diabetes, gall stones, and to relieve asthma. In the Chinese system of medicine, jackfruit is found to be of use in overcoming the influence of alcohol [60]. Depending on the variety, jackfruit was shown to be rich in compounds like carotenoids, volatile acids sterols, tannins, and important compounds like morin, dihydromorin, cynomacurin, artocarpin, isoartocarpin, cyloartocarpin, artocarpesin, oxydihydroartocarpesin, artocarpetin, norartocarpetin, cycloartinone, and artocarpanone [60].

Recently, two new chalcones, artocarpusins A and B; one new flavone, artocarpusin C; one new 2-arylbenzofuran derivative, artocarstilene A, were isolated from the twigs of the plant [64]. Artocarpesin has been found to reduce obesity associated inflammation by suppressing production of nitric oxide and prostaglandin E2 in macrophage cells [65]. Artocarpin has the ability to reduce cell viability in dose-dependent manner, causes alteration in cell morphology, and induces apoptosis of T47D breast cancer cells [55]. Gemichalcones A and artocarpin were presented to have moderate inhibitory activity on the proliferation of the PC-3 and H460 cell line [64]. Methanol extract of* A. heterophyllus* has likewise been showed to be nontoxic to normal cells (HEK293) but toxic to tA549 cell line as determined by MTT and SRB cytotoxic assays [66]. Jacalin, from the seeds, was similarly reported for its anticarcinogenic effect [50]. Jacalin was found to be strongly mitogenic for human CD4^+^ T-lymphocytes. It is considered as an important constituent to be studied for the evaluation of the immune status of patients infected with human immunodeficiency virus HIV-1 [65].

*A. heterophyllus* was also found to inhibit *α*-amylase activity* in vitro* and in rat plasma [67]. In STZ-induced diabetic rats, chronic administration of the ethylacetate fraction of* A. heterophyllus* leaves daily (20 mg/kg) for 5 weeks resulted in a significant lowering of serum glucose (39%), cholesterol (23%) and triglyceride levels (40%) compared to control group. Such results mediated by the extract were comparable with those produced by glibenclamide (0.6 mg/kg) [63]. Pressurized hot water extract of* A. heterophyllus* seeds was observed to possess a significant antiglycation activity [68]. Furthermore, similar investigation into the leaves of* A. heterophyllus* has indicated its efficiency in the attenuation of glycosylation of hemoglobin, enhancement in the transport of glucose across cells, stimulation of insulin release, and inhibition of cholesterol biosynthetic enzymes [63–69]. With regard to the antimicrobial activity, the butanol extracts of the root bark and fruits were found to be active against* Bacillus cereus*,* Bacillus coagulans*,* Bacillus megaterium*,* Bacillus subtilis*,* Lactobacillus casei*,* Micrococcus luteus*,* Micrococcus roseus, Staphylococcus albus*,* Staphylococcus aureus*,* Staphylococcus epidermidis*,* Streptococcus faecalis*,* Streptococcus pneumoniae*,* Agrobacterium tumefaciens*,* Citrobacter freundii*,* Enterobacter aerogenes*,* Escherichia coli*,* Klebsiella pneumonia*,* Neisseria gonorrhoeae*,* Proteus mirabilis*,* Proteus vulgaris*,* Pseudomonas aeruginosa*,* Salmonella typhi*,* Salmonella typhimurium*, and* Serratia marcescens* as evaluated by the disc diffusion method [70].

### 2.4. *Eugenia jambolana *Lam. (Myrtaceae): Jambolan

*Eugenia jambolana*, commonly known as jambolan, black plum, or jamun, is a tropical plant native to India and produces oblong or ellipsoid fruits (berries) annually [71]. The ripe fruit contains around 60 Kcal of energy, water (83.13 g), vitamin A (3 IU), pantothenic acid (0.16 mg), vitamin C (14.3 mg), calcium (19 mg), phosphorus (17 mg), magnesium (15 mg), potassium (79 mg), sodium (14 mg), and iron (16.2 mg) [72]. A glycoside in the seed, jamboline, is considered to have antidiabetic properties. It a is also reported to be a rich source of ellagitannins, including corilagin, 3,6-hexa hydroxyl diphenoyl glucose and its isomer 4,6-hexahydroxy diphenoyl glucose, 1-galloyl glucose, 3-galloyl glucose, gallic acid, and ellagic acid [72]. Decoction of 5 g of grounded seeds in 15 cl of water is prepared against diabetes and taken 3 times daily. Infusion of the bark along with the bark of guava (*Psidium guajava*) can be taken against dysentery and diarrhoea in folk medicines [36]. According to the Unani system of medicine, the fruits are used as liver tonic, to enrich blood and strengthen teeth and gums [71]. In India, juice extracted from the leaves is mixed with cow's milk and taken orally twice a day after taking food for 3 months for control of diabetes. Fresh fruits are taken to get relief from stomach ache, colic, and other digestive complaints [73]. The juice from the stem bark is mixed with butter milk and taken orally every day before going to bed to treat constipation. The same recipe, when taken early in the morning on empty stomach, is claimed to stop blood discharge in faeces [74]. Juice from the bark is also given to women with a history of repeated abortions [75].

The antibacterial potential of the leaf essential oil, petroleum ether, chloroform, ethyl acetate, and methanol extracts of the leaves were studied against human pathogenic bacteria, namely,* Bacillus cereus*,* Enterobacter faecalis*,* Salmonella paratyphi*,* Staphylococcus aureus*,* Escherichia coli*,* Proteus vulgaris*,* Klebsiella pneumoniae*,* Pseudomonas aeruginosa*, and* Serratia marcescens* and were found to have activity quite comparable with the standard antibiotics [76]. Additionally, aqueous extract of leaf of jambolan was shown to be effective against clinical isolates of* Citrobacter *spp.,* Salmonella typhi*,* Salmonella typhimurium*,* Shigella boydii*,* Shigella sonnei*, and* Streptococcus faecalis *[77]. Aqueous, methanolic, and hydromethanolic extracts of the leaf are also effective against cariogenic bacteria such as* Streptococcus mutans*, and to suppress plaque formation* in vitro *[71], whereas aqueous extract of the seed was also found to produce significant and dose dependent antidiarrhoeal, antimotility, and antisecretory effects [78].

The ethanolic extracts of the fruit pulp, kernel, and seed coat were evaluated with gallic acid, quercetin, and trolox as reference molecules. The kernel extract was observed to be better than the seed coat and pulp extracts in 2,2-diphenyl-1-picrylhydrazyl (DPPH), superoxide radical scavenging, and hydroxyl radical scavenging assays [79]. The anthocyanin rich pulp extract was shown to inhibit the iron-induced lipid peroxidation in the rat brain, liver mitochondria, testes, and human erythrocyte cells* in vitro *[80]. Many clinical and experimental studies suggest that different parts of the jambolan especially fruits and seeds possess promising activity against diabetes mellitus [81]. A recent review by Ayyanar et al. [81] has elaborated on the antidiabetic action of various parts of jambolan. Though the exact mechanisms of action in animals are not fully understood, it is suggested that jambolan exerts an action by mimicking sulphonylurea and biguanides. It is postulated that it exerts about its hypoglycaemic action by stimulating of surviving *β* cells of islets of Langerhans to release more insulin. Moreover, it has also been reported to modulate glucose-6-phosphatase content in liver which caused an overall increase in glucose influx. Incubation of the* E. jambolana *aqueous seed extract with rat everted gut sacs resulted in the inhibition of the transport of glucose across the membrane, showing hypoglycemic activities [82]. Jambolan fruit is associated with decreased risk of secondary complications of diabetes. It is reported that oral feeding of fruit extract might help in the prevention of cataract development and decrease the risk of diabetic patients developing atherosclerosis as it contains oleanolic acid. This natural triterpenoid is known for its antioxidant properties by hampering the chemical reactions that generate toxic free radicals and reduces the action of free radicals in atherosclerosis by 60–90% [81].

### 2.5. *Eugenia uniflora *Linn. (Myrtaceae): Pitanga Fruit or Cayenne Cherry

*Eugenia uniflora*, a tropical fruit-bearing shrub, is native to Surinam and widely distributed throughout Brazil. The ripe fruit contains around calcium (9 mg), phosphorus (11 mg), carotene (1200–2000 IU), and ascorbic acid (20–30 mg) but is low in iron (0.2 mg) [83]. The phytochemical screening showed the presence of phenolics eugeniflorin D1 and eugeniflorin D2, tannins, triterpenes, heterosides, anthraquinones, flavonoids, and saponins [66]. The leaves yield essential oil containing citronellal, geranyl acetate, geraniol, cineole, terpinene, sesquiterpenes, and polyterpenes [84]. The Pitanga fruit contains 26 mg/100 g of total anthocyanins and the identification of anthocyanins revealed the presence of cyanidin-3-glucoside and delphinidin-3-glucoside [85]. Infusion of young leaves or young shoots together with leaves of lemongrass (*Cymbopogon citratus*) is prepared to fight against flu and soothe headache. The infusion is also effective against diarrhea [36] and against gingival bleedings [86]. In Nigeria, a decoction of the leaves, mixed with guava (*Psidium guajava*) and neem (*Azadirachta indica*), is taken against fever and gastrointestinal disturbances in infants [87]. Throughout Brazil the ripe fruit is used in popular medicine as a diuretic, antirheumatic, febrifuge, and anti-inflammatory agent and as a therapeutic agent for stomach diseases [88]. In Paraguay, water decoctions are used to lower cholesterol and blood pressure [89]. The essential oil showed antimicrobial activity against two important pathogenic bacteria,* Staphylococcus aureus* and* Listeria monocytogenes*, and against two fungi;* Candida lipolytica* and* Candida Guilliermondii*. It was found to reduce lipid peroxidation in the kidney [88]. Acute oral administration of the essential oil did not cause lethality or toxicological effects in mice. Santos et al. [90] also described that* E. uniflora* presents anti-*Trypanosoma* activity, which might help to combat infectious diseases such as Chagas disease caused by the parasite* Trypanosoma cruzi*. Tannins present were shown to have an inhibitory effect on Epstein Barr Virus DNA polymerase. Epstein Barr Virus is a human B lymphotropic herpes virus which is known to be closely associated with nasopharyngeal carcinoma [55]. Ethanol extract of* E. uniflora *also showed hypoglycemic activity by inhibiting maltase and sucrase activities as well as help decrease plasma triglyceride levels by inhibiting lipase activity [91]. Besides, the essential oil of* E. uniflora* leaves was demonstrated to reduce acetaminophen induced lipid peroxidation and restored all of the biochemical parameters modified by the injury like nonprotein thiol content, *δ*-aminolevulinate dehydratase, and glutathione-S-transferase activities and plasma activities of aspartate aminotransferase and alanine aminotransferase [92].

### 2.6. *Lagenaria siceraria *(Molina) Standley (Cucurbitaceae): Bottle Gourd

*Lagenaria siceraria* has a wide occurrence in India where both its bottle or bell-shaped fruits and aerial parts are consumed as a vegetable [93]. The gourd has low energy value (14 Kcal) and low fat content (0.02 g) [94] but is moderate in vitamin C (10 mg) and water (96%) [95]. The iron content of bottle gourd taken with the peel on is 11.87 mg. It is also a rich source of phosphorus (240 mg), potassium (3320 mg), and magnesium (162 mg) [95].* L. siceraria *is rich in cardiac glycosides, alkaloids, saponins, tannins, and flavonoids [96]. The fruit contains good choline level-a lipotropic factor, known to be important in the treatment of mental disorders. It is also reported to contain triterpenoid cucurbitacins B, D, G, H, and 22-deoxycucurbitacin, the bitter principle of Cucurbitaceae [97]. A decoction of young tender fruits or infusion of young shoots is prepared to fight against diabetes and hypertension (2-3 cups per day), whilst decoction of the seeds is effective for cleaning the digestive system and against constipation [36]. A decoction of the peels is also consumed at a frequency of 1 cup for 3 days for the treatment of diabetes [98]. The pulp of the fruit is considered to have cooling effects, diuretic, and antibilious properties and is effective against cough, fever, asthma, and other bronchial disorders. Decoction of leaves of* L. siceraria *is taken against jaundice, while decoction of skin of young fruits is documented to be taken against uremia, albuminuria, inflammation, and diabetes [36]. A novel protein, langenin, was isolated from seeds and reported to have antitumor, antiviral, antiproliferative, and anti-HIV activities [99]. Langenin has been documented to be a ribosome-inactivating protein (RIP). RIPs catalytically cleave N-glycosidic bonds of adenine in a specific RNA sequence, resulting in inhibition of protein synthesis. The potential applications of RIPs include conjugation with antibodies to form immunotoxins for cancer therapy [100]. Hydroalcoholic extract of* L. siceraria* indeed showed strong and dose-dependent inhibition of cancer cell line of the lung (A549) [101].

Juice extract of the fresh fruits of* L. siceraria *on rats caused significant decrease in the levels of serum cholesterol and triglyceride levels by inhibition of lipoprotein lipase and lecithin-cholesterol acyl-transferase activity making triglycerides available for uptake and metabolism by tissues. Serum biochemistry changes suggested that the juice extract has a tonic effect on the kidneys and the liver and these organs play a central role in drug metabolism [96]. The juice extract also shows potential diuretic activity comparable to that of furosemide (20 mg/kg) and might be important in management of hypertension [98].* L. siceraria *fruit powder also shows cardioprotective effects on isoprenaline-induced toxicity [99]. Deshpande et al. [102] studied antihyperglycemic activity of* L. siceraria* fruit in rats with induced hyperglycemia by alloxan monohydrate. It was found that the extract effectively prevented biochemical changes of induced hyperglycemia. Similar results were also obtained in a more recent study [93], where methanol extracts of aerial parts of* L. siceraria* reduced significantly fasting blood glucose levels and improved the antioxidant and histologic observations of the pancreas, kidney, and liver in STZ-induced diabetic rats. Katare et al. [103] have studied the administration of freshly prepared bottle gourd juice (200 mL) to human participants on empty stomach for 90 consecutive days. A notable reduction in blood glucose levels along with a significant reduction in total cholesterol (17.8%), serum triglycerides (16–22%), andLDL-c (22.2%) and a considerable decrease in VLDL-c was observed in diabetic subjects on juice therapy. Juice administration also contributed to significant elevations in superoxide dismutase and catalase activities in both diabetic (40.5%) and normal healthy subjects. Additionally,* L. siceraria* fruit extract was shown to ameliorate fat amassment and serum TNF- in high-fat diet-induced obese rats [104].

### 2.7. *Momordica charantia* Linn. (Cucurbitaceae): Bitter Melon

*Momordica charantia* or bitter melon, also known as balsam pear or karela, is a valuable vegetable worldwide and has been extensively used in folk medicine as a remedy against diabetes. Bitter melon is grown in tropical areas of Asia, the Amazon, East Africa, the Caribbean, and throughout South America [105]. The nutrient profile of* M. charantia* has been listed by Jeyadevi et al. [106] and Yuwai et al. [107]. The fruit (per 100 g) has an energy value of 60 Kcal and contains approximately 23 mg calcium, 171 mg potassium, 2.4 mg sodium, 119.92 mg magnesium, 5.97 mg iron, 38 mg phosphorus, 96 mg vitamin C, and 126 mg *β*-carotene. Depending on the varieties ranging from the wild type to the hybrid green or hybrid white, bitter gourd is documented to have negligible level of reducing sugar and a total amount of protein ranging from 1.17 to 2.4% [108].

Examination of the phytochemicals of this plant indicates the presence of various active components like momorcharins, momordenol, momordicilin, momordicins, momordicinin, momordin, momordolol, charantin, charine, cryptoxanthin, cucurbitins, cucurbitacins, cucurbitanes, cycloartenols, diosgenin, elaeostearic acids, erythrodiol, galacturonic acids, gentisic acid, goyaglycosides, goyasaponins, and multiflorenol [109]. In India it is used for abortions, birth control, constipation, diabetes, eczema, fat loss, food, fever, gout, hemorrhoids, hydrophobia, hyperglycemia, increasing milk flow, intestinal parasites, jaundice, kidney stones, leprosy, liver, menstrual disorders, pneumonia, psoriasis, rheumatism, snakebite, and vaginal discharge [110]. A decoction is prepared by adding bitter melon leaves to 2 glasses of water. Diabetic people traditionally consumed 1/3 cup of the decoction thrice a day for effective control of blood sugar level [110]. Indeed, various scientific studies validated that fresh juice of bitter melon can lower blood sugar values and keep insulin level under check. Hypoglycemic activity has been documented to be due to the mixture of phytoconstituents isolated and the charantins which are insulin-like peptides [111]. Polypeptide-p is one of the few active compounds in bitter melon which has been extensively studied [112]. Several possible modes of the hypoglycemic actions of* M. charantia* have been proposed. These include stimulation of peripheral and skeletal muscle glucose utilization, inhibition of intestinal glucose uptake, inhibition of adipocyte differentiation, suppression of key gluconeogenic enzymes, stimulation of key enzyme of hexose monophosphate pathway, enhancement of insulin secretion by the Islets of Langerhans, and regeneration and preservation of islet *β* cells and their functions [113]. Supplementation of STZ-induced diabetic rats with* M. charantia *extract indeed showed improved activity level of enzymes hepatic glucokinase, hexokinase, glucose-6-phosphatase, and glycogen synthase [114]. Acute intravenous administrations of whole-plant of* M. charantia* extract produced dose-dependent, significant reductions in systemic arterial blood pressure, and heart rates of both normal and hypertensive Dahl salt-sensitive rats [115]. A more recent study stated that* M. charantia* can also inhibit the proliferation of preadipocytes in a dose dependent manner [116]. Furthermore, the clinical potential of bitter melon has been examined in several human cell line experiments. Momordin increases the expression of peroxisome proliferator-activated receptor (PPAR) *δ* mRNA in hepatoblastoma cells (HepG2), which is important in the regulation of glucose metabolism and fatty acid storage. Bitter melon juice also has been reported to significantly reduce sterol regulatory element-binding protein 1c (SREBP-1c) when applied to primary preadipocytes, further highlighting the prospective benefit of this compound in glycemic control [112]. Moreover, owing to its free radical scavenging abilities, it showed hepatoprotective effects against xenobiotics [117] like cyclophosphamide, a chemotherapy drug [118]. Though the exact mechanisms need to be elucidated,* M. charantia *has also been shown to decrease serum/tissue lipid parameters in hyperammonemic rats [119]. High levels of ammonia are considered toxic, affecting the central nervous system by causing functional disturbances that could even lead to coma and death [119]. It was found that the levels of serum and tissue cholesterol, triglycerides, free fatty acids, and phospholipids were significantly increased in ammonium chloride-induced hyperammonemic rats. However, upon administration of ethanolic extract of* M. charantia *fruit all these changes were significantly restored to almost normal levels. Several phytochemicals isolated from* M. charantia *like alpha- and beta-momorcharin, lectin, and* Momordica *anti-HIV protein (MAP30) have been documented to have* in vitro* antiviral activity against Epstein-Barr, herpes, HIV, coxsackie virus B3, and polio viruses [120]. MAP30 is reported to be a ribosomal inactivating protein that inhibits the HIV-1 reverse transcription, integration, and syncytium formation between the infected and the new white blood cells. MAP30 also inhibits the viral core protein synthesis [120]. Hexane, ethyl acetate, and ethanol seed extracts also exhibited broad spectrum antimicrobial activity against* Escherichia coli*,* Candida albicans*,* Staphylococcus aureus*,* Staphylococcus epidermidis*, and* Klebsiella pneumonia* [121].

### 2.8. *Moringa oleifera* Lam. (Moringaceae): Drumstick Tree

*Moringa oleifera*, also known as drumstick tree or horseradish tree, is a pan-tropical species that is native to the sub-Himalayan tracts of India, Pakistan, Bangladesh, and Afghanistan. It has long been used in Ayurvedic and Unani systems of medicines [122]. Many parts of this plant including the leaves, pods, and flowers are edible and are used as a highly nutritive vegetable and have even been used to combat malnutrition [123]. Moringa has even been advocated as “natural nutrition for the tropics” [124]. In folk medicine, leaves are used as purgative, applied as poultice to sores, rubbed on the temples for headaches, against fevers, sore throat, bronchitis, eye, and ear infection. Leaf juice is reported to control glucose levels and is applied topically to reduce glandular swelling. In contrast, the juice from the root bark is put into ears to relieve ear aches [125]. In the Philippines, it is known as “mother's best friend” for its virtue of increasing breast milk production and is often recommended for anemia [126]. Moringa leaves have been reported “ounce for ounce” to contain more *β*-carotene than carrots, more calcium than milk, more iron than spinach, and more potassium than bananas, high quality proteins, and even more vitamin C than oranges [124]. Melo et al. [127] reported that* M. oleifera* leaves contained good level of protein (22.75 g), fiber (7.92 g), and soluble carbohydrates (51.66 g) per 100 g dry weight. Equally, a previous study [128] showed that the dried leaves had crude protein levels of 30.3% and 19 amino acids. Different mineral contents were also identified like calcium (3.65%), phosphorus (0.3%), magnesium (0.5%), potassium (1.5%), sodium (0.164%), sulphur (0.63%), zinc (13.03 mg/kg), copper (8.25%), manganese (86.8 mg/kg), iron (490 mg/kg), and selenium (363 mg/kg). Additionally, 17 fatty acids were found among which *α*-linolenic acid made up 44.57%.

Regarding the medicinal value of leaves of* M. oleifera*, it was described to have antihypertensive, anti-inflammatory, and antimicrobial effects due to the presence of specific components like 4-(4′-*O*-acetyl-*α*-L-rhamnopyranosyloxy)benzyl isothiocy-anate, 4-(*α*-L-rhamnopyranosyloxy) benzyl isothiocy-anate, niazimicin, pterygospermin, benzyl isothiocyanate, and 4-(*α*-L-rhamnopyranosyloxy) benzyl glucosinolate [129]. Isolated compounds, like niazinin and niazimicin, showed blood pressure lowering effect in rats, possibly through calcium antagonist effect. Niazimicin has been as well stated to be chemoprotective in nature [125]. The alkaloid, morigine, isolated from seed extract has the ability to relax bronchioles which is beneficial in respiratory disorders [129].

Drumstick leaves also showed decrease in blood glucose level in STZ-mild induced diabetic rats in a dose-response relationship [130]. Similarly, treatment of methanol extracts of* M. oleifera* pods in STZ-induced diabetic albino rats resulted in significant reduction in the advancement of diabetes. There was a significant reduction in serum glucose and nitric oxide as well as parallel increases in serum insulin, protein levels, and a reduction in the degenerative changes in the *β* cells [131].* M. oleifera* has also been described as a promising food plant in protecting the liver against acetaminophen-induced liver injury via restoration and elevation of glutathione level in the liver [132]. Hannan et al. [133] demonstrated that ethanol extract of* M. oleifera* leaf promoted neurite outgrowth in a concentration-dependent manner and significantly promoted the earlier stages of neuronal differentiation. Therefore, this subsequently increased the number and length of dendrites, the length of axon, and the number and length of both dendrite and axonal branches and facilitated synaptogenesis. Thus, this extract shows potential application in neuronal survival. Findings from another study [134] tend to suggest that the ethanol extract of* M. oleifera* leaves possesses CNS depressant and anticonvulsant activities. This might possibly be mediated through the enhancement of central inhibitory mechanism involving release *γ*-amino butyric acid (GABA) and thus supports its traditional use against epilepsy. Hydroalcoholic extract of* M. oleifera *leaves also displayed the potential to improve or prevent vascular intimal damage and atherogenesis and thus help in limiting cardiovascular complications [135]. For instance, oral administration of the extract (100 and 200 mg/kg/body weight) showed significant reduction in elevated levels of body weight, total cholesterol, triglycerides, low density lipoprotein, very low density lipoprotein, and parallel significant increase in high density lipoprotein level. Intravenous administration of the hydroalcoholic extract delayed the plasma recalcification time in rabbits and also inhibited ADP induced platelet aggregation* in vitro*, which was comparable to commercial heparin. Pertaining to antimicrobial activity, the aqueous and ethanolic extracts of the leaves showed high activity against* Candida albicans *and Gram positive bacteria such* Staphylococcus aureus *and* Enterococcus faecalis *but weak activity for Gram-negative bacteria such as* Escherichia coli*,* Salmonella typhimurium*,* Klebsiella pneumoniae*, and* Pseudomonas aeruginosa* [136]. Almost similar results were obtained from another study which tested the acetone extract of* M. oleifera* [137].

### 2.9. *Punica granatum *Linn. (Punicaceae): Pomegranate

*Punica granatum,* commonly known as pomegranate, granada (Spanish), and grenade (French), is native from Iran to and Northern India. It was cultivated and naturalized over the whole Mediterranean region since ancient times [138] and has been used in several systems of medicine for a variety of ailments. In the Ayurveda system of medicine, almost all parts (fruits, flowers, seeds, leaves, and bark) have been used for remedial purposes. For instance, it has been used as an antiparasitic agent and a blood tonic, against aphthae, diarrhea, and ulcers. In addition in the Unani system, it is used for the management of diabetes. It is also reported that decoction prepared with the bark and water in a ratio of 1 : 5 is recommended against intestinal worms. A decoction of the bark, cinnamon (*Cinnamomum verum*), and cloves (*Syzygium aromaticum*) is taken against diarrhea [36].* Punica granatum *was referred to as “a pharmacy unto itself” [139]. The proximate nutritional value of raw pomegranate fruit per 100 g is as follows: water (77.93 g), protein (1.67 g), carbohydrate (18.70 g), total lipid (1.17 g), fibre (4 g), calcium (10 mg), iron (0.3 mg), magnesium (12 mg), and phosphorus (36 mg) [140]. Pomegranate aril juice provides about 16% of an adult's daily vitamin C requirement per 100 mL serving and is a good source of vitamin B5 (pantothenic acid) and potassium [139].

Phytochemical isolation and characterization revealed different functional components in various parts of the plant [141]. The pomegranate leaf extract was found to contain tannins like punicalin, pedunculagin, gallagic acid, and ellagic acid. The flowers are composed of ursolic acids, triterpenoids like oleanolic acid, maslinic acid, and asiatic acid. The pomegranate seeds are a rich source of conjugated fatty acids such as linoleic acid and linolenic acid and other lipids such as punicic acid, stearic acid, palmitic acid, and phytosterols. The juice is known to be a rich source of antioxidants from the polyphenols, tannins, anthocyanins, coenzyme Q10, and lipoic acid [141]. Studies have also looked at the beneficial effects of pomegranates antioxidant activity* in vivo* and* in vitro*. Bekir et al. [142] indeed confirmed the high level of total phenolics, flavonoids, and anthocyanins in the methanol extract of* P. granatum* leaves and also demonstrated good DPPH and ABTS radical scavenging potential and strong lipoxygenase inhibition activities. It was reported that pomegranate juice and seed extracts have 2-3 times the antioxidant capacity of either red wine or green tea [143]. Aviram et al. [144] analyzed* in vivo* and* in vitro *antiatherogenic properties of pomegranate fruit parts, that is, the peel, arils, seeds, and flower. All extracts were shown to possess antioxidative properties* in vitro*. After consumption of pomegranate juice, peels, arils, and flowers by e-deficient mice, the atherosclerotic lesion area was significantly decreased, as compared to placebotreated group. The pomegranate fruit extract consumption resulted in lower serum lipids and glucose levels by 18% to 25%. The uptake rates of oxidized-LDL by e-deficient-peritoneal macrophages were significantly reduced by approximately 15%. Pomegranate juice consumption causes a decrease in procarcinogen activation through CYP activity/expression (CYP1A2 and CYP3A) [145] and protects rat gastric mucosa from ethanol or aspirin toxicity [146]. Pomegranate fruit extract was shown to inhibit cell growth through modulations in the cyclin kinase inhibitor-cyclin-dependent kinase system, followed by apoptosis of highly aggressive human prostate carcinoma PC3 cells. These events were associated with alterations in the levels of Bax and Bcl-2 shifting the Bax: Bcl-2 ratio in favour of apoptosis [147].

Pomegranate rind extract has also been shown to have topical anti-inflammatory and analgesic properties since it dose-dependently attenuated the inflammatory responses in acute and chronic models of inflammation [148]. The anti-inflammatory and analgesic effects were achieved through inhibiting the leukocyte in filtration and modulating the proinflammatory cytokines IL-*β* and TNF-*α* [148]. The anti-inflammatory and antimicrobial properties of pomegranate were furthermore assessed through a randomized controlled clinical trial which evaluated the effectiveness of mouth rinse with pomegranate and chamomile plant extracts, against chlorhexidine 0.12% in the gingiva bleeding condition [149]. It was found that the gingival bleeding index in the groups of patients with gingivitis and chronic periodontitis, who used pomegranate extract mouthwash, was as statistically significant as those observed in groups of chamomile and chlorhexidine 0.12%. The antimicrobial activity was confirmed by the study of Abdollahzadeh et al. [150]. The latter demonstrated that methanol extract of* P. granatum* peel could inhibit growth of certain pathogens involved in oral infections like* Streptococcus mutans*,* Staphylococcus aureus*,* Streptococcus salivarius*,* Streptococcus sanguinis*,* Staphylococcus epidermidis*,* Actinomyces viscosus*,* Lactobacillus acidophilus*, and* Candida albicans*.

### 2.10. *Solanum nigrum *Linn. (Solanaceae): Black Nightshade

*Solanum nigrum *usually grows as a weed in moist land and is semicultivated in a few countries in Africa and Indonesia. It is mainly utilized as a vegetable. The leaves have an energy value of 38 Kcal, calcium (99–442 mg), phosphorus (75 mg), iron (1–4.2 mg), and ascorbic acid (20 mg) but is also high in oxalate (around 58–90 mg) [151]. It has been found that* S. nigrum *contains glycoalkaloids such as solamargine, solasonine and solanidine, saponins, and glycoprotein, exhibiting antitumor activity [152]. In South India, fresh leaves cooked with onion bulbs and cumin seeds are taken against stomach ache. Cooked leaves are also recommended in cases of hypotension, anaemia, and to improve vision. Juice of fresh leaves, mixed with honey, is applied on painful lesions which appear on the buccal mucosa [153]. The juice may also be applied on burns. One handful of the leaves is boiled in 1L of water and used to reduce fever [36]. The leaves are used as poultice for rheumatic and gouty joints. Leaves are also used in dropsy, nausea, and nervous disorders [151]. Decoction of the plant depresses the CNS and reflexes of the spinal cord. The whole plant is used as anti-inflammatory, expectorant, cardiotonic, digestive, diuretic, and laxative, for swelling, cough, and asthma. The plant is also effective against, haemorrhoids, nephropathy, ophthalmopathy, and general weakness [154]. Leaf decoction is given to drink once a day on an empty stomach for 7 days to manage stomach ulcers [155]. The water extract of* S. nigrum *shows protective effects against carbon tetrachloride induced chronic liver damage in rats and this hepatoprotective effect might be contributed to its modulation on detoxification enzymes and its antioxidant and free radical scavenger effects [156]. Aqueous plant extracts possess antiproliferative activity as demonstrated by growth inhibition of cervical carcinoma. The antiproliferative activity of solanine on transformed cell lines* in vitro *is mainly due to its ability to facilitate induction of apoptosis by inhibiting Bcl-2, an antiapoptotic protein [157].* S. nigrum *also exhibits antiulcer activity through acid and peptic suppression in aspirin induced ulcerogenesis in rats [151].

Other compounds like solasonine, *β*1-solasonine, solamargine, and solanigroside P were also proved to have cytotoxicity to MGC-803 cells. It was established that compounds with three sugar units and *α*-L-rhamnopyranose at C-2 or a hydroxyl group on the steroidal backbone may be potential candidates for the treatment of gastric cancer. The mechanism of action may be related to the decrease of mutation p53, the increase of the ratio of Bax to Bcl-2, and the activation of caspase-3 to induce apoptosis. More specifically, solamargine induced an accumulation of cells in S phase with an increasing apoptotic rate, which indicated that solamargine induced apoptosis perhaps through S phase arrest [158]. However, another study revealed that solamargine could inhibit the growth of human hepatoma SMMC-7721 and HepG2 cells by inducing cell cycle arrest at the G2/M phase [159]. The petroleum extract of* S. nigrum* berries was tested for its therapeutic potential against asthma. The extract demonstrated ability to inhibit clonidine-induced catalepsy and increased leukocyte and eosinophilic count due to milk allergen significantly. It also showed maximum protection against mast cell degranulation caused by clonidine and demonstrate presence of antiasthmatic compound, *β*-sitosterol [160]. Results from the study of Sohrabipour et al. [161] showed that administration of 1 g/l of* S. nigrum* fruit extract to drinking water of diabetic animals for 8 weeks can decrease blood glucose levels. However the exact mechanism whereby this is accomplished is not well understood. Intraperitoneal glucose tolerance test revealed that* S. nigrum* extract leads to improvement in glucose intolerance in condition of chronic diabetes. The outcomes tend to suggest that* S. nigrum* may repair pancreas *β* cells and enhance insulin secretion or increase Glu4 translocation in the cell membrane, which then decreases blood glucose levels.* S. nigrum *also decreased calcium/magnesium ratio which might indicate a reduced atherogenic risk since Ca/Mg ratio is a marker of vascular tone, in which an increase represents increased vascular reactivity [161].

## 3. Discussion

Medicinal foods are the oldest form of therapies known to mankind and the traditional pharmacopeia of various populations has documented such practice [9, 162]. Various countries possess rich endemic flora and a rich milieu of biodiversity that still remain an untapped reservoir for prototype molecules or pharmacophores for the pharmaceutical industry. For instance, according to Gurib-Fakim [162], about 25% of the global biodiversity in plants are found in Indian Ocean Islands and Sub-Saharan Africa and represent key constituents in the traditional medicinal systems in this region, especially in rural areas. The common use of medicinal food plants to treat and/or manage diverse health problems, ranging from acute cold and flu, stress, and pain to more severe chronic illnesses has led to the term “phytotherapy” to describe such practices. The medicinal foods are often perceived by the patient community as being safe since they have been used by communities in various countries for generations and some are still being commonly consumed today [163]. With such notion there has been a steady growth of the world market for the functional foods and phytopharmaceuticals—the pharmaceuticals designed using traditional compounds derived from botanicals instead of chemicals [164]. Indeed, the emergence of this new market segment called “Health and Wellness” has provided potential benefits to consumers' diet and new business opportunities for producers [165] such that it is now known as the fastest growing food sector with a compound annual growth rate of 8.6% in the last 10 years to 2012 and a global value of US$ 625 billion in 2012 [166]. Even though such success of the functional food market and trade has been attributed to various factors like research-oriented collaborative networks or the onset of “industrial marriage,” which is the joint efforts in sharing of resources and skills for functional food product development by pharmaceutical and food manufacturers, consumer acceptance remained the decisive factor in positive market response [166]. The challenge brought forward is that consumers want to eat healthily, with low calories and nutritionally added value but without missing the enjoyment and pleasure of eating from any modified food texture and quality [167]. Consequently, it seems clear that a collaborative work between food researchers, food technologists, nutritionists, and food designers might be crucial in the design of the functional food product especially in maintaining the bioavailability and functionality of added active ingredients. Currently, efforts are still being made in understanding the physiological or behavioural interactions between medicinal foods and pharmaceuticals, their pharmacokinetics, and pharmacodynamics. This still offers a challenge to the scientific community due to the complexity of plant matrix and bioactive molecules. Little is known about the processes through which the metabolites are absorbed into the body, reach their biological target, and are eliminated [168]. The risks for food-drug or nutrient drug interactions also exist. An interaction is considered significant from a clinical perspective if it alters the therapeutic response. Food-drug interactions can result in 2 main clinical effects: the decreased bioavailability of a drug, which predisposes to treatment failure, or an increased bioavailability, which increases the risk of adverse events and may even precipitate toxicities [168].

Many of the food plants presented here show very promising medicinal properties, but attention should be drawn on other issues such as possible food-drug interactions. For instance, studies highlighted the possible interaction between pomegranate juice and warfarin [169, 170]. Pomegranate juice was shown to inhibit cytochrome P450 enzymes involved in warfarin metabolism. Concomitant use of* M. charantia* with oral hypoglycaemic medication was also analysed.* M. charantia* was demonstrated to augment the hypoglycaemic effect of rosiglitazone, a PPAR-gamma agonist in STZ induced diabetic rats [171]. The enhanced hypoglycemic effect was also observed with combination of metformin and* M. charantia* juice compared when the drug alone was administered [172]. Thus, it can be suggested that* M. charantia* might produce synergistic effect when used along with hypoglycaemic drugs which may be either beneficial or harmful to patients and should be used under careful supervision.

The possibility of biotransformation which occurs in gastrointestinal tract or through hepatic metabolism in the patient should not be ruled out or ignored. This point is critically important in assessing the therapeutic benefits of purified compounds. This is because the active ingredient may not be present in the initial medicinal food but is only produced after absorption and metabolic transformation [163]. In this framework, current developments in the area of functional food design and technology have already been made to demonstrate that the bioavailability of the bioactive components can be improved by the proper selection and development a delivery and protection system such as use of microparticles. Microencapsulation is a process by which the functional ingredient (core) is packaged within a secondary material (encapsulant) to form a microcapsule (2 to 2000 *μ*m) [173]. The encapsulant in the form of matrix or shell forms a protective coating around the core, isolating it from its surrounding environment (pH, water activity, time, pressure, physical force, or enzymatic action) until its release is triggered by changes in its environment [173]. This avoids undesirable interactions of the bioactive with other food components or chemical reactions that can lead to degradation of the bioactive, with the possible undesirable consequences on taste and odour as well as negative health effects [174]. Çam et al. [175] have reported the effects of microencapsulation conditions on product quality of pomegranate peel phenolics and found that addition of microencapsulated pomegranate peel phenolics showed significant improvement of the antioxidant and *α*-glucosidase inhibitory activities of the enriched ice creams compared with control sample. Besides, more than 75% of panelists involved in the study positively rated the phenolic enriched ice creams in sensory evaluation, which lends supports to such products for commercial introduction to the general public with the potential as functional food. This technology can be a decisive step forward towards solving problems regarding the targeting of active functional ingredients to its target site and controlling its delivery rate to a specific organ or tissue [175]. However, a more recent technological extension of microencapsulation involves the formation of active loaded particles with diameters ranging from 1 to 1000 nm, a process known as nanoencapsulation. Indeed, currently the second largest area of nanotechnology application is in the food sector [176]. For instance, application of nanoencapsulation has been studied for various functional food ingredients. Despite curcumin's multiple medicinal benefits, low bioavailability of curcumin continues to be highlighted as a major challenge in developing formulations for clinical efficacy [176]. This is because it has been found that low serum and tissue levels of curcumin are observed irrespective of the route of administration due to extensive intestinal and hepatic metabolism and rapid elimination thus restraining curcumin's bioavailability [177]. Moreover, curcumin is insoluble in water and degrades at neutral to basic pH conditions and found to be photosensitive and requires careful handling. In this view, nanoparticles have been developed where unlike free curcumin was shown to readily disperse in aqueous media and demonstrated comparable* in vitro* therapeutic efficacy to free curcumin against a panel of human pancreatic cancer cell lines [176]. Nevertheless, the toxicological aspect and risk assessment for such technologies are still unclear and should not be overlooked [177]. It is possible that such nanomaterial upon degradation of the structure to release the active ingredient will form compounds with other food material, interact with one another, solubilize on reaction with acid or digestive enzyme, or remain in a free state, while in the alimentary canal and how this will affect absorption of other nutrients is unknown [177].

Likewise, despite that plant bioactive constituents have proved to offer various health benefits, their potential risks should also be considered. Typically, ethnopharmacological surveys are used to describe uses, dosages, sources, and methods of preparation of traditional medicines of food plants. Nevertheless, their application in examining the adverse effects, authenticity, quality, contraindications, and other safety aspects of these preparations remains limited and should not be overlooked. Cravotto et al. [178] reported that about 12% of the plants currently on the Western market have no substantial published scientific studies on their properties, while about 1 in 200 were toxic or allergenic, such that their use ought to be discouraged or forbidden. The quality of medicinal foods can be affected by several factors both intrinsic and extrinsic. Species differences and seasonal variations are examples of intrinsic factors that can affect the qualitative and quantitative accumulation of the biologically active chemical constituents produced and accumulated in the plant [179]. Extrinsic factors include environmental conditions, agricultural practices, postharvest handling, storage, manufacturing, inadvertent contamination, substitution, and intentional adulteration [179]. Hence, continued use of medicinal foods for disease management presents risks of potential adverse effects, especially when being used in combination with synthetic drugs. Few plants have been subjected to randomized clinical trials under the International Conference on Harmonization (ICH) Good Clinical Practice Guidelines to determine their efficacy and safety [180].

Another challenge is legislation governing the trade and marketing of the medicinal or functional foods [181]. Individual countries have their own legislation on functional foods. While in USA, functional foods are regulated by the FDA and also encompass dietary supplements; in the E.U, there is no legislation operating on functional foods and there is no legal authority for its definition [181]. In order to protect public health, it was deemed essential to guarantee that such novel foods or functional food ingredients are subjected to a safety assessment through a community procedure before they are placed on the market along with a regulation on nutrition and health claims [181]. Health-related claims should be scientifically substantiated, valid for the food as it is consumed or in its future anticipated use to reach the minimal effective dose and communicated clearly, understandably, and truthfully to the consumer [181]. It is the European Food Safety Authority (EFSA) that has the objective of providing scientific opinion on the safety of food plants and its bioactive constituents. EFSA even requires RCT (Randomized Controlled Trial) giving proof of beneficial physiological effects of a compound on a healthy population. However, one problem which arises is that RCT might be of help only when purified compounds are analyzed but subsequently become practically impossible using whole food plants, where a plethora of bioactive compounds exist, which have different physiological targets. Also, phytochemical compositions vary greatly in hydrophilic and lipophilic nature and in different plant parts. Thus, traditional knowledge still remains an important aspect that should not be neglected at the expense of emerging science and technologies. It still remains crucial for safety assessment of functional foods in the E.U. countries where there are no proper legislations because it indicates us which parts of the plants were traditionally used, how the preparation is made (infusion, decoction or alcoholic extracts), and what are the specific conditions of use [181].

## 4. Conclusion

Medicinal foods have long been integrated in the cultural and habitual dietary pattern of various populations. Research has demonstrated that nutrition plays a crucial role in the prevention of chronic disease and now with the recognition that typical foods may provide prophylactic benefits, efforts are being directed towards promoting the “functional diet.” The new concept of functional foods has been identified as a promising field to boost nutritional sciences to the forefront of preventive medicines for both existing and emerging diseases of man. However, the exact mechanisms of actions of isolated compounds of various traditionally used plant extracts still remain to be elucidated in many cases. The use of medicinal food plants as dietary adjuncts among patients on conventional pharmacological therapy should be carefully assessed due to possibility of food-drug interactions or herb-herb interactions. Hence, combined approaches of parallel preclinical studies involving* in vitro*,* in vivo* and* in silico* models and well-designed clinical studies are crucial to provide basic toxicological data to assess its suitability in this regard. The present review also draws attention on some active metabolites of the plants with the potential for new drugs development or improved plant medicines. The elucidation of the mechanisms of action of biologically active extracts and phytocompounds should be strengthened and given priority in future clinical investigations. In this view use of medicinal foods could provide phytotherapy a new dimension and enable their use to treat and/or manage diseases which have hitherto been treated using synthetic drugs alone with limited therapeutic window.
